# Impact of micronutrient supplementation during pregnancy on birth weight, duration of gestation, and perinatal mortality in rural western China: double blind cluster randomised controlled trial

**DOI:** 10.1136/bmj.a2001

**Published:** 2008-11-07

**Authors:** Lingxia Zeng, Yue Cheng, Shaonong Dang, Hong Yan, Michael J Dibley, Suying Chang, Lingzhi Kong

**Affiliations:** 1 Department of Public Health, Xi’an Jiaotong University College of Medicine, PO Box 46, Xi’an, Shaanxi 710061, China; 2 School of Public Health, University of Sydney, Room 307A, Edward Ford Building (A27), University of Sydney, NSW 2006, Australia; 3 George Institute for International Health, PO Box M201, Sydney, NSW 2050, Australia; 4 Chinese Centre for Disease Control and Prevention, Beijing 100050, China; 5 Bureau for Disease Prevention and Control, Ministry of Health, Beijing 100044, China

## Abstract

**Objective** To examine the impact of antenatal supplementation with multiple micronutrients or iron and folic acid compared with folic acid alone on birth weight, duration of gestation, and maternal haemoglobin concentration in the third trimester.

**Design** Cluster randomised double blind controlled trial.

**Setting** Two rural counties in north west China.

**Participants** 5828 pregnant women and 4697 live births.

**Interventions** Villages were randomised for all pregnant women to take either daily folic acid (control), iron with folic acid, or multiple micronutrients with a recommended allowance of 15 vitamins and minerals.

**Main outcome measures** Birth weight, length, and head circumference measured within 72 hours after delivery. Neonatal survival assessed at the six week follow-up visit.

**Results** Birth weight was 42 g (95% confidence interval 7 to 78 g) higher in the multiple micronutrients group compared with the folic acid group. Duration of gestation was 0.23 weeks (0.10 to 0.36 weeks) longer in the iron-folic acid group and 0.19 weeks (0.06 to 0.32 weeks) longer in the multiple micronutrients group. Iron-folic acid was associated with a significantly reduced risk of early preterm delivery (<34 weeks) (relative risk 0.50, 0.27 to 0.94, P=0.031). There was a significant increase in haemoglobin concentration in both iron-folic acid (5.0 g/l, 2.0 to 8.0 g/l, P=0.001) and multiple micronutrients (6.9 g/l, 4.1 to 9.6 g/l, P<0.001) groups compared with folic acid alone. In post hoc analyses there were no significant differences for perinatal mortality, but iron-folic acid was associated with a significantly reduced early neonatal mortality by 54% (relative risk 0.46, 0.21 to 0.98).

**Conclusion** In rural populations in China antenatal supplementation with iron-folic acid was associated with longer gestation and a reduction in early neonatal mortality compared with folic acid. Multiple micronutrients were associated with modestly increased birth weight compared with folic acid, but, despite this weight gain, there was no significant reduction in early neonatal mortality. Pregnant women in developing countries need sufficient doses of iron in nutrient supplements to maximise reductions in neonatal mortality.

**Trial registration** ISRCTN08850194.

## Introduction

Neonatal deaths contribute greatly to child mortality in developing countries, and these deaths have steadily increased as a percentage of all deaths of children under the age of 5.^1^ ^2^ In China, by 2004 neonatal mortality accounted for more than half of all deaths in children under 5.^3^ Low birthweight babies are at higher risk of morbidity and mortality than those of normal birth weight^4^ and are also at risk of postnatal growth retardation, with possible adverse long term effects on their physical and cognitive development.^5^ ^6^ One of the major causes of low birth weight in developing countries is the poor nutritional status of the mother before and during pregnancy, resulting in restricted fetal growth especially during the third trimester.^7^

Poor quality diet and inadequate intake, combined with increased nutrient requirements for placental and fetal growth, can lead to multiple micronutrient deficiencies in pregnancy and contribute to higher rates of low birth weight.^8^ To address these multiple deficiency states Unicef has proposed the use of multiple micronutrient supplements in pregnancy that provide the recommended individual intakes for pregnant women.^9^

China is the most populated developing country in the world, and the prevalence of low birth weight varies across different socioeconomic groups and areas. A 1999 survey of low birth weight in China showed a prevalence of low birth weight of 7.6% and 11.8% in “poor” and “very poor” counties, respectively.^10^ With an average prevalence of low birth weight of 5.9%, an estimated 1.2 million low birthweight babies are born in China every year.

The only antenatal supplement promoted by the Ministry of Health in China is folic acid to prevent congenital neural tube defects.^11^ There are no specific policies or programmes for the distribution of multiple micronutrient or iron-folic acid supplements during pregnancy, even to disadvantaged women. To provide evidence in China to formulate public health policy on nutrient supplementation in pregnancy we conducted a community based cluster randomised controlled trial in a disadvantaged rural population. We assessed the impact of iron-folic acid and multiple micronutrient supplements during pregnancy, compared with folic acid alone, on birth anthropometry, duration of gestation, and maternal haemoglobin concentration in the third trimester. We also conducted a post hoc assessment of the impact of the supplements on perinatal mortality, comprising stillbirths and early neonatal deaths.

## Methods

### Experimental design

The trial took place in two poor rural counties in Shaanxi Province of north west China. A pilot study found a low birth rate of 13% and 57% of women with anaemia (haemoglobin <110 g/l) in the third trimester. Because of the scale of this trial we allocated the same treatment to all pregnant women in a given village. In 2001 in the first county there were 14 townships and 234 villages, and in the second county there were 20 townships and 327 villages. Randomisation of villages was stratified by county with a fixed ratio of treatments (1:1:1) and blocking of 15 to ensure geographical balance with an approximately equal distribution of treatments per township.

The randomisation schedule was generated off site with a pseudo-random number generator in SAS version 6 (SAS Institute, Cary, NC). A treatment colour code was assigned to each village based on the treatment allocation schedule. The treatment codes were opened only once all data had been collected and blinded analysis of the primary hypothesis was completed.

### Study population and sample size

The study sample consisted of all women resident in the counties who became pregnant between August 2002 and January 2006 and who fulfilled trial selection criteria (see below). Before the start of the trial, we estimated that we needed a sample of 2255 eligible pregnant women per treatment to detect a 25% reduction in low birth weight between either iron-folic acid or multiple micronutrient and folic acid (control) groups, assuming a two tailed test, with α=0.05 and β=0.20, and a prevalence of low birth weight of 9.3% (based on a three month sample in 2000 of births from a county hospital in the study area). We increased the sample size to 2480 pregnant women per group to account for 10% fetal loss and loss to follow-up.

At the start of the trial we also expected that recruitment would take 2.5 years, based on registered live births in the counties in the previous year. Recruitment was slower than expected, however, because many births were in women who lived in large cities in eastern China but who returned to their village to give birth and register their child. After 3.5 years the trial sponsor requested that recruitment be stopped because of limitations with funding. At that stage, January 2006, we had recruited and randomised 5828 eligible women. We estimated that this sample of about 1900 women per treatment would provide 80% power to detect a 50 g difference in birth weight between either iron-folic acid or multiple micronutrients and folic acid (control) groups, assuming a two tailed test, with α=0.05 and birth weight standard deviation (SD) of 436 g (based on values observed across all treatment groups). This sample size, however, only had 80% power to detect a 40% reduction in low birth weight between treatment groups, assuming a two tailed test, with α=0.05 and a prevalence of low birth weight of 4.6% (prevalence observed across all treatment groups). It also had a similarly low power of 80% to detect a 50% reduction in early neonatal mortality between treatment groups, assuming a two tailed test, with α=0.05 and an early neonatal mortality rate of 15 per 1000 live births. We did not adjust for the cluster randomised design in any of these estimates of sample size.

### Enrolment and pregnancy surveillance procedures

Village doctors, with support from the township maternal and child healthcare workers, recruited women by active surveillance for pregnancy in women of reproductive age. At the start the village doctor conducted a mini-survey of all women of reproductive age living in their village to identify those who were likely to become pregnant, including newly married women, those not using contraception, or those who said they wanted a child. Trained village doctors obtained informed verbal consent for pregnancy monitoring, and consenting women were visited every month and asked about the date of their last menstrual period. Women with periods delayed by more than five days had a urine pregnancy test, and confirmed pregnancies were reported to the township maternal and child health worker. Pregnancies in women resident in the studied townships or counties were passively detected at antenatal clinics in local health facilities. If the diagnosed pregnancy was less than 28 weeks’ gestation, trained MCH staff from township hospitals sought informed verbal consent to participate in the trial from the woman and her partner.

Newly identified pregnant women were interviewed to record their sociodemographic status and their menstrual, reproductive, medical, and family history. Recruited pregnant women received three free antenatal care checks, at which they were asked about pregnancy complications and underwent a physical examination including blood pressure and weight measurement. Haemoglobin was measured at the third antenatal check. Trained MCH staff, with monitoring by project staff, collected and recorded all the information collected during the pregnancy until the six week follow-up visit in a pregnancy care record book, which served both as a clinical record and data capture instrument.

### Interventions

Villages were randomly assigned for women to receive one of three daily antenatal supplements: multiple micronutrients, iron-folic acid, or folic acid alone (control). The multiple micronutrient supplements were formulated to contain approximately the WHO/Unicef recommended dietary allowances for each of 15 minerals or vitamins as follows: 30 mg iron, 400 µg folate, 15.0 mg zinc, 2.0 mg copper, 65.0 µg selenium, 150.0 µg iodine, 800.0 µg vitamin A, 1.4 mg vitamin B-1 (thiamine), 1.4 mg vitamin B-2 (riboflavin), 1.9 mg vitamin B-6, 2.6 µg vitamin B-12, 5.0 µg vitamin D, 70.0 mg vitamin C, 10.0 mg vitamin E, and 18.0 mg niacin.^12^ Iron-folic acid supplements contained 60 mg iron (twice the amount of elemental iron in the multiple micronutrients) and 400 µg folic acid. The folic acid supplement contained 400 µg of folic acid. The three supplement types were produced by Beijing Vita Nutritious Products, Beijing, China, and were of identical appearance and packaged in blister packs.

At enrolment, each woman received a blister pack containing 15 capsules with instructions to take one capsule daily. The village doctor visited the women every two weeks to provide more supplements and to retrieve the used blister strips and record the number of remaining capsules. The number of supplements consumed throughout the trial was summed to estimate compliance.

### Measurement of outcomes

Hospital nursing staff measured birth weight within one hour of delivery. In the six county hospitals and the three largest township hospitals (78% of birth weights) birth weight was measured with an electronic scale (type BD 585, Tanita, Dongguan, Guangdong Province, China) with precision to the nearest 10 g. For births in the 31 smaller township hospitals (10% of birth weights) and for home births (12% of birth weights) birth weight was measured with a baby scale (type RTZ-10A-RT, Wuxi Weigher Factory, Wuxi, China) with precision to the nearest 50 g. For home deliveries, township maternal and child health staff visited the women at home within 72 hours of delivery to measure the baby and gather information on delivery. We excluded from analyses newborn anthropometry collected later than 72 hours after birth. Low birth weight was defined as <2500 g. Birth length was measured to the nearest 1 mm by using a portable measuring board with fixed head piece. Occipitofrontal head circumference was measured with a tape to the nearest 1 mm.

Gestational age at birth was measured as completed days based on the first day of the last menstrual period, obtained at the baseline interview. Preterm delivery was defined as delivery before 37 completed weeks’ gestation and early preterm delivery as before 34 weeks. Small for gestational age babies were defined as those whose weight was below the 10th centile of the gestational age-sex specific US reference for fetal growth.^13^

Maternal haemoglobin concentration was measured in capillary blood collected early in the third trimester (gestation 28-32 weeks) from a subsample of 599 pregnant women with a birth outcome, who were consecutively enrolled from 6 July 2004 to 28 October 2005. HemoCue portable spectrophotometers (Ängelholm, Sweden) were used to assay haemoglobin concentration and were calibrated daily and all measurements were made within the temperature operating range of this device (15-30°C). We excluded haemoglobin measurements collected before 6 July 2004 because in winter these measurements were taken below the operating range, potentially resulting in incorrect values. Anaemia in the third trimester was defined as haemoglobin <110 g/l.^14^

Village doctors or hospital staff reported fetal losses during pregnancy, birth outcome, delivery information, and neonatal and maternal deaths; maternal and child health staff recorded data with precoded structured forms. Neonatal survival was assessed at the six week follow-up visit.

We defined perinatal deaths as stillbirths (fetuses delivered at 28 weeks’ gestation or later with no signs of life and recorded as occurring before the onset of or during labour) plus early neonatal deaths (deaths among liveborn infants occurring within seven days of delivery). Neonatal deaths were defined as deaths among liveborn infants occurring within 28 days of delivery. Project staff re-interviewed all women who had a stillbirth or neonatal death to check the reported information and, for hospital or clinic deliveries, to cross check with medical records.

### Statistical analysis

To assess the effectiveness of randomisation we examined the baseline characteristics of the clusters and the individual pregnant women across treatment groups. A wealth index was constructed from an inventory of 16 household assets or facilities with a principal component analysis method,^15^ and this index was categorised into thirds as an indicator for the poorest, middle income, and richest households. The mean number of supplements consumed and treatment compliance rates (percentage of days that supplements were consumed) were also examined.

All analyses were conducted using the intention to treat principle. We included only liveborn infants in the analyses of birth anthropometry because most stillborn infants were not measured. We estimated mean differences and 95% confidence intervals for birth weight and gestation and adjusted for the effect of randomisation by villages using generalised estimating equation linear models with an independent correlation structure, which is a suitable modelling strategy where there are more than 40 clusters per treatment group.^16^ ^17^ The adjusted mean differences in birth weight, birth length, head circumference, and gestation, and their 95% confidence intervals, were computed relative to the folic acid group. Similarly, we calculated adjusted mean differences for newborn anthropometry and gestation at birth and their 95% confidence intervals to compare the multiple micronutrient and the iron-folic acid groups. To adjust for cluster randomisation, we applied generalised estimating equation binomial regression models with log link and exchangeable correlation structures to estimate the relative risks and 95% confidence intervals for low birth weight, small for gestational age, preterm delivery, and anaemia with the folic acid group as the reference and with multiple birth indicator as a cofactor, and similarly to compare the multiple micronutrient and iron-folic acid groups.

We analysed perinatal mortality only in singleton births to prevent bias from the effect of multiple births, which have higher mortality risk. We calculated rates of stillbirth and perinatal death using the number of pregnancies at 28 weeks’ gestation as the denominator. Neonatal death rates were calculated with the number of live births as the denominator. Kaplan-Meier survival analysis was used to estimate the survival probabilities of liveborn singleton infants from birth to 28 days and to compare survival across treatment groups. To adjust for the cluster randomisation, we applied generalised estimating equation binomial regression models with log link and exchangeable correlation structures to estimate the relative risks and 95% confidence intervals for stillbirth and perinatal and neonatal death rates with the folic acid group as the reference. Wald χ^2^ tests assessed the overall effects of the three treatments for each outcome. We used Stata version 9.2 (Stata/SE 9.2 StataCorp, College Station, TX) for all analyses.

## Results

Figure 1 shows the flow of participants through the trial. Enrolment began on 1 August 2002 and continued until 9 July 2005; the last baby born to a trial participant was on 24 January 2006, and all women completed the trial by 28 February 2006. Over the 3.5 year period, there were 7144 confirmed pregnancies from the monitored population: 727 women refused to participate in the trial, 498 did not meet the inclusion criteria, and 91 did not participate for other reasons (fig 1). We enrolled and randomised 5828 women, but of these 133 women were lost to follow up, 279 stopped taking supplements and refused to continue to participate, and 601 had a spontaneous or induced abortion or other medical condition resulting in fetal loss. There were a total of 4650 pregnancies that resulted in at least one live birth. There were 167 stillbirths, where birth weight was not usually recorded. There were a total of 4697 live births; birth weight was missing or was measured beyond 72 hours after birth in 276. There were 4421 (94%) live births with birth weight available for analysis and 222 perinatal deaths. The final row of boxes in figure 1 shows the numbers included in the intention to treat analyses for each of the trial outcomes examined. Across all treatment groups, 7% of the women were enrolled in a village other than their usual place of residence, and this was usually their mother’s village.

**Figure fig1:**
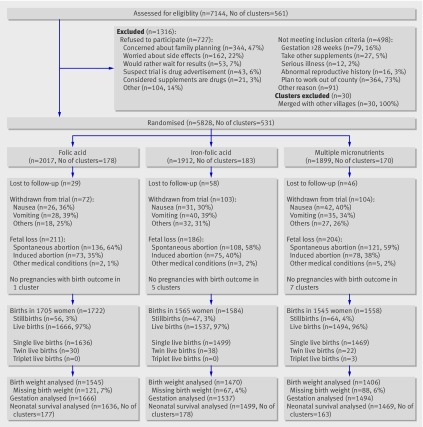
**Fig 1** Participant flow chart

The sociodemographic characteristics and the anthropometric measurements at enrolment and the cluster and individual level baseline characteristics were balanced by treatment groups (table 1).[Table tbl1] The reproductive history of the pregnant women was similar across groups and reflected China’s “one child policy,” with 3585 (61.5%) women having their first pregnancy (table 2).[Table tbl2] The percentage of women delivering at home was balanced across treatment groups with 9.4% (149) for iron-folic acid group, 12.4% (193) for the multiple micronutrients group, and 11.4% (195) for the folic acid only group. There were 47 pairs of twins and one set of triplets. These multiple births were not balanced across the treatment groups, with 19 pairs of twins (of 1584 births) in the iron-folic acid group, 12 pairs of twins and one set of triplets (of 1558 births) in the multiple micronutrient group, and 17 pairs of twins (of 1722 births) in the folic acid group (P=0.031).

**Table 1 tbl1:** Baseline characteristics of clusters, households, and participants by treatment group. Figures are numbers (percentages) unless stated otherwise

	Folic acid	Iron-folic acid	Multiple micronutrients
No of clusters	178	183	170
Mean (SD) population in clusters in 2001	826 (502)	781 (434)	890 (515)
Mean (SD) births in clusters in 2001	6.8 (5.4)	6.5 (4.4)	7.6 (8.6)
Mean (SD) pregnancies/cluster/year	6.3 (1.1)	6.3 (1.1)	6.0 (1.3)
Mean (SD) No of family members/cluster	4.2 (0.77)	4.2 (0.81)	4.2 (0.89)
Maternal age (years):			
Mean (SD)	24.8 (4.4)	24.8 (4.3)	24.8 (4.4)
15-19	152 (7.5)	153 (8.0)	160 (8.4)
20-24	950 (47.1)	875 (45.8)	867 (45.7)
25-29	564 (28.0)	567 (29.7)	555 (29.2)
30-34	312 (15.5)	277 (14.5)	276 (14.5)
≥35	39 (1.9)	40 (2.1)	41 (2.2)
Women’s education:			
<3 years	133 (6.6)	100 (5.3)	118 (6.2)
Primary	527 (26.3)	495 (26.0)	542 (28.6)
Secondary	1087 (54.2)	1022 (53.7)	962 (50.8)
High school and above	259 (12.9)	288 (15.1)	271 (14.3)
Women’s occupation:			
Farmer	1677 (83.9)	1563 (82.3)	1599 (84.8)
Other	322 (16.1)	337 (17.7)	287 (15.2)
Household wealth index:			
Mean (SD)	−0.03 (1.49)	0.07 (1.53)	−0.03 (1.55)
Poorest third	665 (33.0)	600 (31.4)	671 (35.3)
Middle third	725 (35.9)	639 (33.4)	627 (33.0)
Richest third	627 (31.1)	673 (35.2)	601 (31.7)

**Table 2 tbl2:** Baseline characteristics at enrolment related to pregnancy by treatment group. Figures are numbers (percentages) unless stated otherwise

	Folic acid	Iron-folic acid	Multiple micronutrients
No of pregnant women	2017	1912	1899
Mean (SD) No of pregnancies	1.6 (0.7)	1.6 (0.7)	1.6 (0.7)
Parity:			
0	1223 (60.6)	1184 (61.9)	1178 (62.0)
1	714 (35.4)	669 (35.0)	657 (34.6)
≥2	80 (4.0)	59 (3.1)	64 (3.4)
Gestation (weeks):			
Mean (SD)	13.8 (5.8)	13.6 (5.6)	13.9 (5.6)
<12	884 (43.8)	835 (43.7)	820 (43.2)
12-15	432 (21.4)	424 (22.2)	424 (22.3)
16-28	701 (34.8)	653 (34.2)	655 (34.5)
Height (cm):			
No of women	1998	1897	1878
Mean (SD)	158.8 (5.1)	158.9 (5.2)	158.7 (5.3)
Weight (kg):			
No of women	1970	1864	1846
Mean (SD)	52.5 (6.0)	52.7 (6.4)	52.7 (6.1)
BMI (kg/m^2^):			
No of women	1968	1863	1840
Mean (SD)	20.8 (2.2)	20.9 (2.3)	20.9 (2.2)
Mid-upper arm circumference (cm):			
No of women	1983	1884	1872
Mean (SD)	23.1 (2.1)	23.2 (2.2)	23.2 (2.1)

There was a high level of compliance with the supplementation in all treatment groups (table 3).[Table tbl3] The mean number of doses of supplements consumed per women during pregnancy was 165, and this was similar in each group. About 6% of women consumed fewer than 90 supplements during the pregnancy, and over 80% consumed more than 120 supplements.

**Table 3 tbl3:** Compliance* and number of doses of supplements consumed by treatment group

	Folic acid	Iron-folic acid	Multiple micronutrients
No who gave birth	1705	1565	1545
No (%) who complied:			
0-25	9 (0.6)	16 (1.0)	18 (1.2)
26-50	24 (1.4)	29 (1.9)	32 (2.1)
51-75	95 (5.6)	110 (7.0)	88 (5.7)
76-100	1577 (92.5)	1410 (90.1)	1407 (91.1)
Mean (SD)	93.4 (12.7)	91.9 (14.8)	92.6 (14.9)
No (%) of supplements consumed:			
<90	105 (6.2)	85 (5.4)	106 (6.9)
91-119	181 (10.6)	160 (10.2)	154 (10.0)
120-179	684 (40.1)	638 (40.8)	630 (40.8)
≥180	735 (43.1)	682 (43.6)	655 (42.4)
Mean (SD)	165 (44)	166 (44)	165 (45)

*Compliance calculated by number of actual supplements consumed divided by number of supplements expected to be consumed.

### Impact of nutrient supplementation on anthropometry and gestation at birth

The intracluster correlation coefficient for birth weight was 0.03 (95% confidence interval 0.015 to 0.052). There was no evidence of an effect of iron-folic acid on mean birth weight (P=0.17) but birth weight was significantly higher (42 g, 7 to 78 g, P=0.019) in the multiple micronutrients group compared with the folic acid alone (table 4).[Table tbl4] The increase in birth weight observed in the iron-folic acid and the multiple micronutrient groups corresponded with reductions in the risk of low birth weight (<2500 g) compared with folic acid alone of 19% (P=0.20) and 22% (P=0.14), respectively. In a post-hoc analysis of babies who were small for gestational age, there were no significant differences in the proportion across the three treatment groups, although the proportion in the multiple micronutrient group was slightly lower (table 4).[Table tbl4]

**Table 4 tbl4:** Birth anthropometry, gestation, and haemoglobin, with adjusted difference or relative risk for comparison of multiple micronutrients or iron-folic acid with folic acid alone

Birth outcomes	No (%*) of infants	Mean (SD)	Adjusted† difference or relative risk (95% CI)	P value
**Birth weight (g)‡**				
Folic acid	1545	3153.7 (444.9)		
Iron-folic acid	1470	3173.9 (424.4)	24.3 (−10.3 to 59.0)	0.169
Multiple micronutrients	1406	3197.9 (438.0)	42.3 (7.1 to 77.5)	0.019
**Birth weight <2500 g**				
Folic acid	82 (5.3)	—		
Iron-folic acid	66 (4.5)	—	0.81 (0.59 to 1.12)	0.199
Multiple micronutrients	57 (4.1)	—	0.78 (0.56 to 1.08)	0.139
**Small for gestational age**				
Folic acid	280 (18.1)	—		
Iron-folic acid	278 (18.9)	—	1.04 (0.89 to 1.22)	0.618
Multiple micronutrients	238 (16.9)	—	0.95 (0.82 to 1.12)	0.549
**Gestation at birth (weeks)**				
Folic acid	1666	39.63 (1.93)		
Iron-folic acid	1537	39.84 (1.69)	0.23 (0.10 to 0.36)	0.001
Multiple micronutrients	1494	39.82 (1.80)	0.19 (0.06 to 0.32)	0.004
**Preterm <37 weeks**				
Folic acid	102 (6.1)	—		
Iron/folic acid	76 (4.9)	—	0.79 (0.58 to 1.07)	0.131
Multiple micronutrients	78 (5.2)	—	0.86 (0.64 to 1.14)	0.285
**Early preterm <34 weeks**				
Folic acid	30 (1.80)	—		
Iron-folic acid	15 (0.98)	—	0.50 (0.27 to 0.94)	0.031
Multiple micronutrients	19 (1.27)	—	0.70 (0.38 to 1.30)	0.259
**Birth length (cm)§**				
Folic acid	1367	48.8 (2.9)		
Iron-folic acid	1299	49.1 (2.7)	0.24 (0.02 to 0.46)	0.032
Multiple micronutrients	1251	49.1 (2.8)	0.22 (−0.05 to 0.49)	0.117
**Head circumference (cm)¶**				
Folic acid	1350	33.1 (1.6)		
Iron-folic acid	1278	33.2 (1.6)	0.11 (−0.06 to 0.28)	0.207
Multiple micronutrients	1234	33.1 (1.6)	0.02 (−0.16 to 0.21)	0.8
Haemoglobin** (g/l)				
Folic acid	218	105.3 (14.2)		
Iron-folic acid	193	110.1 (14.8)	5.0 (2.0 to 8.0)	0.001
Multiple micronutrients	188	111.8 (14.1)	6.9 (4.1 to 9.6)	<0.001
**Anaemia¶ (<110 g/l)**				
Folic acid	133 (61.0)	—		
Iron-folic acid	87 (45.1)	—	0.74 (0.61 to 0.91)	0.003
Multiple micronutrients	81 (43.1)	—	0.72 (0.59 to 0.88)	0.001

*If applicable.

†Adjusted for multiple births and cluster randomisation in general estimating equation linear models.

‡Data missing for 121 in folic acid, 67 in iron-folic acid, 88 in multiple micronutrients.

§Data missing for 299 in folic acid, 239 in iron-folic acid, 243 in multiple micronutrients.

¶Data missing for 316 in folic acid, 260 in iron-folic acid, 260 in multiple micronutrients.

**In subsample of 599 pregnant women with birth outcome who were consecutively enrolled from 6 July 2004 to 28 October 2005.

The intracluster correlation coefficient for gestation at birth was 0.02 (0.004 to 0.036). Table 4 shows there was a significant increase in the duration of gestation of 0.23 weeks (0.10 to 0.36 weeks, P=0.001) in the iron-folic acid group and 0.19 weeks (0.06 to 0.32 weeks, P=0.004) in the multiple micronutrient group compared with folic acid.[Table tbl4] The increase in the mean duration of gestation in the iron-folic acid group corresponded with a 21% reduction in the risk of preterm delivery (<37 weeks) (P=0.13) and a significant 50% reduction in the risk of early preterm delivery (<34 weeks) (relative risk 0.50, 0.27 to 0.94, P=0.031). Compared with folic acid alone, the increase in the mean duration of gestation observed in the multiple micronutrient group corresponded with non-significant reductions in the risk of preterm delivery (<37 weeks) and early preterm delivery (<34 weeks) of 14% (P=0.29) and 30% (P=0.26), respectively (table 4).[Table tbl4]

The intracluster correlation coefficient for birth length was 0.03 (0.007 to 0.047) and for head circumference was 0.08 (0.050 to 0.101). Table 4 shows that in the iron-folic acid group there was a significant increase in birth length (0.24 cm; 0.02 to 0.46 cm, P=0.03) compared with folic acid alone. There was no significant effect of iron-folic acid on mean head circumference (P=0.21).There were no significant effects of the multiple micronutrients on either mean birth length (P=0.12) or mean head circumference (P=0.80).

Compared with folic acid alone, the difference in mean birth weight, adjusted for multiple births, gestation at delivery, and cluster randomisation, was 12.6 g for the iron-folic acid and 31.0 g for the multiple micronutrients, indicating that the extension of the duration of gestation contributed to a similar increment in mean birth weight for iron-folic acid (11.7 g) and multiple micronutrients (11.3 g) compared with the folic acid.

### Impact of nutrient supplementation on maternal haemoglobin concentration

Haemoglobin samples were available for 599 women in the third trimester. The baseline characteristics for these women were balanced across the treatment groups, and there were no significant differences between the women with and without haemoglobin measurements. Also there was no significant difference in the mean gestation at haemoglobin testing (P=0.54), which was 32.0 weeks (SD 2.6), 31.9 weeks (SD 2.8), and 32.2 weeks (SD 3.1) for the folic acid, iron-folic acid, and multiple micronutrient groups, respectively. There was a significant increase in haemoglobin concentration with both iron-folic acid and multiple micronutrients compared with folic acid (P=0.001 and P<0.001, respectively). In both the iron-folic acid and the multiple micronutrient groups, however, more than 40% of the women were still anaemic in the third trimester (table 4).[Table tbl4]

### Impact of supplementation on perinatal mortality

Post hoc analyses showed no significant impact on the risk of perinatal mortality (stillbirths and early neonatal mortality) either in the iron-folic acid (P=0.307) or in multiple micronutrients group (P=0.336) compared with folic acid (table 5).

**Table 5 tbl5:** Mortality outcomes (in singleton births)

	Folic acid			Iron-folic acid			Multiple micronutrients			Relative risk (95% confidence interval)*	
	No in group	Rate/1000		No in group	Rate/1000		No in group	Rate/1000		Iron-folic acid *v* folic acid	Multiple micronutrients *v* folic acid
Pregnancies with single live or stillbirth:											
Total	1688	—		1546	—		1532	—		—	—
Stillbirths (≥ 28 weeks)	52	30.8		47	30.4		63	41.1		1.01 (0.67 to 1.51)	1.39 (0.95 to 2.04)
Live births	1636	—		1499	—		1469	—		—	—
All neonatal deaths	33	20.2		16	10.7		18	12.3		0.53 (0.29 to 0.97)	0.61 (0.34 to 1.10)
Early neonatal deaths	24	14.7		10	6.7		15	10.2		0.46 (0.21 to 0.98)	0.71 (0.36 to 1.39)
Perinatal deaths	76	45.0		57	36.9		78	50.9		0.84 (0.59 to 1.19)	1.18 (0.85 to 1.63)

*Adjusted for cluster randomisation with general estimating equation binomial model.

Post hoc analyses also showed no effect of iron-folic acid compared with folic acid on the stillbirth rate, though there was a non-significant increase of 39% (relative risk 1.39, 0.95 to 2.04) in the multiple micronutrients group. When we combined all fetal losses (spontaneous abortions and stillbirths), the rates were 103.1/1000 pregnancies for folic acid, 93.7/1000 for iron-folic acid, and 111.3/1000 for multiple micronutrients, indicating no evidence of an altered risk for total fetal losses with iron-folic acid (relative risk 0.91, 0.74 to 1.12) or with multiple micronutrients (1.15, 0.94 to 1.40) compared with folic acid.

The post hoc analyses showed a reduction in the risk of early neonatal mortality among infants born to women randomised to receive either iron-folic acid or multiple micronutrients compared with folic acid. The magnitude of the reduction (54%) was significant for the iron-folic acid group (relative risk 0.46, 0.21 to 0.98) compared with folic acid but not for the multiple micronutrients group (29%; 0.71, 0.36 to 1.39).

Figure 2 illustrates cumulative mortality curves for infants from birth to 28 days, with lower mortality rates for neonates whose mothers received iron-folic acid and multiple micronutrients. Most of the divergence of the mortality curves occurred in the first seven days after birth, after which the curves were about parallel. The overall differences were of borderline significance (P=0.055), but the difference between iron-folic acid and folic acid alone was significant (P=0.032). The other individual treatment comparisons (multiple micronutrients *v* folic acid and multiple micronutrients *v* iron-folic acid) were not significant (P=0.077 and P=0.708, respectively).

**Figure fig2:**
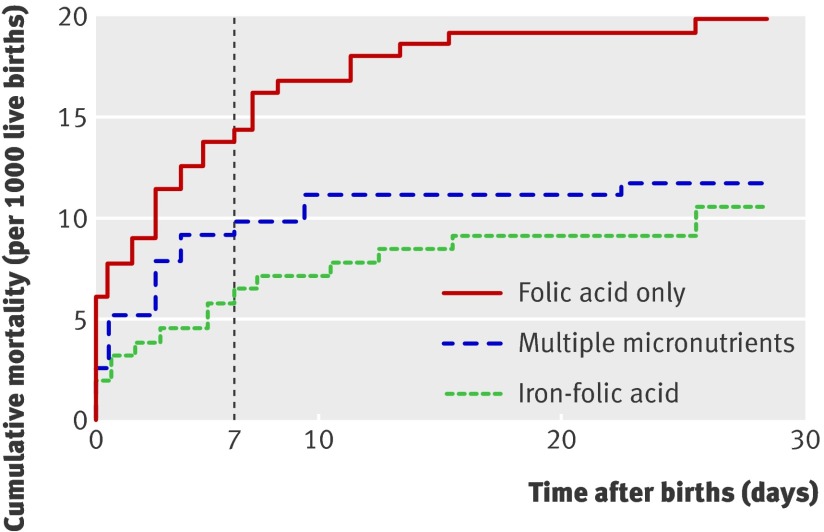
**Fig 2** Cumulative mortality curves for infants from birth to 28 days by treatment. Log rank test of difference: P=0.055 for three groups; 0.032 for iron-folic acid *v* folic acid; 0.077 for multiple micronutrients *v* folic acid; 0.708 for multiple micronutrients *v* iron-folic acid

There were 73 neonatal deaths, including three pairs of twins. The highest number of neonatal deaths because of preterm delivery was in the folic acid group (11/73, 15.1%) with similar numbers for iron-folic acid (5/73, 6.8%) and multiple micronutrients (6/73, 8.2%) groups. There was also a higher number of deaths from birth asphyxia in the folic acid group (8/73, 11.0%) with the same number in the iron-folic acid (3/73, 4.1%) and multiple micronutrient (3/73, 4.1%) groups.

There were no significant differences between multiple micronutrients compared with iron-folic acid for birth anthropometry, duration of gestation, haemoglobin concentration, or perinatal mortality.

On the basis of our results, the number of women who would need to be treated with iron-folic acid from early in pregnancy would be 6.2 (6.1 to 6.3) to prevent one case of maternal anaemia in the third trimester, 83.3 (83.28 to 83.32) to prevent one preterm delivery <37 weeks, 122 (121.99 to 122.01) to prevent one early preterm delivery <34 weeks, and 125 (124.99 to 125.01) to prevent one early neonatal death. With multiple micronutrients the numbers needed to treat would be 5.6 (5.5 to 5.7), 111.1 (111.08 to 111.12), 188.7 (188.69 to 188.71), and 222 (222.19 to 222.21) respectively.

## Discussion

In this trial the response to nutrient supplementation in pregnancy varied by outcome. Compared with folic acid alone, multiple micronutrient supplements were significantly associated with increased birth weight, while iron-folic acid had no significant effect. Iron-folic acid supplements were significantly associated with increased birth length. There was a significant increase in the mean duration of gestation in both the iron-folic acid and multiple micronutrients groups compared with folic acid. In post hoc analyses, the iron-folic acid and the multiple micronutrient supplements had no significant effect on perinatal mortality, but there was a significant 54% reduction in early neonatal mortality in women who received supplementation with iron-folic acid compared with folic acid alone, but no significant effects on stillbirths. Both multiple micronutrients and iron-folic acid were associated with significantly increased maternal haemoglobin concentration in the third trimester compared with folic acid, although nearly half of the women in these groups remained anaemic. Overall, we have shown that antenatal nutrient supplementation is associated with increased maternal haemoglobin concentration in late pregnancy, mean birth weight, and duration of pregnancy and reduced preterm delivery and early neonatal mortality.

The effects of iron-folic acid and multiple micronutrients on the duration of gestation and neonatal mortality seem to be related to the iron in these supplements. We tested the impact of two different doses of iron in pregnancy—namely, 60 mg in the iron-folic acid supplements and 30 mg in the multiple micronutrient supplements. The largest impact on neonatal mortality was with the supplement with the highest dose of iron, and we saw a similar pattern for duration of gestation and effects on preterm delivery. In contrast, a recently reported large scale community based trial from Indonesia found no significant differences for neonatal mortality between daily antenatal supplements with iron-folic acid and multiple micronutrients (WHO/Unicef formulation as tested in China), which both had the same dose of iron (30 mg).^18^ These findings from Indonesia suggest that the higher neonatal mortality rate we observed in China with multiple micronutrients compared with iron-folic acid cannot be explained by the additional micronutrients but by the different dose of iron in the supplements.

In our trial, the multiple micronutrient supplements were associated with a significant increase in birth weight compared with folic acid, but this did not translate into significant reductions in neonatal mortality. Rather the reduction in neonatal mortality seemed to be related to the increased duration of gestation and the reduced number of early preterm deliveries because of iron in the antenatal supplements. Evidence of an important role of antenatal iron supplements in reducing preterm delivery has been reported from the United States.^19^ In the US trial, antenatal iron (30 mg), was associated with a reduced rate of preterm delivery (8% *v* 14%, P=0.05) and increased mean birth weight (108 g, P=0.03). Longitudinal studies in Brazil^20^ and Bangladesh^4^ indicate that preterm infants have much higher neonatal mortality than babies with retarded intrauterine growth or those born at term. In Bangladesh, a study of birth outcomes among low birthweight infants reported that 75% of the neonatal deaths were related to preterm delivery.^4^ These observational findings suggest a plausible reason as to why the increased mean duration of gestation and reduced preterm delivery (especially early preterm delivery) we observed could be related to a large reduction in neonatal mortality.

### Study strengths and limitations

This study was a double blind cluster randomised controlled trial design with a balanced distribution of confounders across treatment groups. Even though randomisation was by village clusters, there were a large number of clusters (531) with about 180 per treatment group and a relatively small number of births in each cluster. Population recruitment of pregnancies allowed complete tracing of fetal losses, including spontaneous and induced abortions and stillbirths, and pregnancy outcomes in both hospital and home deliveries. We used appropriate statistical methods in the data analysis that adjusted for the cluster randomisation.

The location of the study in a socioeconomically disadvantaged area of western China allowed us to examine effects of nutrient supplements in a population with the highest rates of low birth weight and neonatal mortality in the country. Only 2.3% of the enrolled women were lost to follow-up, and 94% of the liveborn infants were weighed within 72 hour of delivery. There was no evidence of under-enumeration of neonatal deaths, with neonatal mortality rates in the folic acid group (20.2/1000 live births) similar to those reported for equivalent rural counties from the national maternal and child health surveillance system (22.5/1000 live births).^3^

The trial was adequately powered to detect small changes in infant anthropometry at birth and the duration of gestation, but one limitation was the low power to detect changes in the prevalence of low birth weight, preterm delivery, and perinatal mortality. The mortality analyses were post hoc rather than a primary hypothesis and we did not have enough data to fully examine the effects of the different supplements on mortality. For example, the non-significant reduction in neonatal mortality associated with multiple micronutrients might have been significant with a larger sample. The outcome data were collected by the local health service staff, although the research team did provide special training on how to measure and record these outcome indicators for the trial.

The balance of treatments by area and therefore by service providers reduced the likelihood of any systematic differences in misclassification of stillbirths and early neonatal deaths by treatment group. The duration of gestation was measured by asking the mother to recall the date of her last menstrual period. The heightened awareness and knowledge about reproduction because of the one child policy in China, however, might have improved the accuracy of the respondent’s recall of her last menstrual period and other perinatal events.

### Comparison with other studies

Our results help to explain the apparent increase in neonatal and perinatal mortality reported from other trials using the same or similar formulation of multiple micronutrient supplements compared with standard iron-folic acid supplements.^21^ ^22^ ^23^ In two trials from Nepal multiple micronutrient supplementation was associated with a non-significant increase in neonatal and perinatal mortality compared with iron-folic acid.^21^ ^22^ In a pooled analysis of these trials neonatal mortality (relative risk 1.52, 1.03 to 2.25) and perinatal mortality (1.36, 1.02 to 1.81) were significantly higher in the multiple micronutrients group compared with iron-folic acid.^23^ These findings were interpreted by the authors as an increased risk of perinatal mortality with multiple micronutrient supplements. Our findings showed a significant reduction in neonatal mortality for iron-folic acid (47%) and a non-significant reduction for multiple micronutrients (39%) compared with folic acid. The greater effect on mortality of iron-folic acid compared with multiple micronutrients could account for why comparisons of multiple micronutrients with iron-folic acid give the appearance of an increased risk of neonatal mortality.

There are similarities between our results and the results of other earlier controlled trials of iron supplementation in pregnancy.^24^ ^25^ In Niger, a double blind randomised controlled trial assessed the impact of 100 mg iron or placebo in the third trimester on iron status of the mother and newborn and on the anthropometric status of the newborn.^24^ The iron supplements were associated with significantly decreased maternal iron deficiency at delivery and at three months after delivery and increased the iron status of the infants at 3 months. Birth length was significantly higher and there was a non-significant increase in birth weight in the iron supplemented group. There were seven fetal and neonatal deaths in the placebo group and one in the iron supplemented group. In the US, a trial of iron supplementation (30 mg/day) versus placebo from 11 weeks’ gestation in pregnant women who were not anaemic reported a significantly longer duration of gestation (0.6 weeks) and fourfold reduction in the risk of preterm low birth weight in the iron supplemented women.^25^ Our findings were also consistent with those from observational studies that reported an increased risk of preterm delivery in women with iron deficiency anaemia in the first trimester of pregnancy.^26^ ^27^ ^28^ ^29^ ^30^ For example, an analysis of perinatal data from Shanghai showed a greater than twofold increase in the risk of low birth weight and preterm delivery in women who had moderate anaemia (90-99 g/l) and a greater than threefold increase for women with severe anaemia (<90 g/l) in the first trimester.^29^

The absorption of iron during pregnancy is a complex physiological process, with increasing relative absorption as gestation progresses but with a decrease in the percentage of the dose absorbed as the total dose increases.^31^ In our trial, even though the amount of iron in the iron-folic acid supplement was twice the amount in the multiple micronutrient supplement this would not necessarily have led to twice the absorption of iron and therefore twice the dose of iron. The vitamin C in the multiple micronutrient supplements might have enhanced the absorption of iron and also contributed to reducing the difference in the absolute amount of iron absorbed. The response of haemoglobin concentration to the iron-folic acid and multiple micronutrients was similar, but the associated neonatal mortality seemed to be proportionate to the likely relative amount of iron contained in each supplement.

We observed a larger birth weight response in the multiple micronutrients group than in the iron-folic acid group. This finding was similar to that observed in other trials comparing iron-folic acid and multiple micronutrients, but the total increase in birth weight in our multiple micronutrients group compared with iron-folic acid group was less than that reported in other studies.^22^ ^32^ ^33^ This did not translate into lower neonatal mortality, although it implies improved fetal growth and development, which might confer other health benefits to the newborn, such as improved infant growth, boosted immune responses, and enhanced cognitive development.

We have shown that micronutrient supplements have important health effects for the newborn infant, with a potential to increase birth weight and birth length through increased duration of gestation and direct effects on fetal growth. Reduced neonatal mortality seems to be a response to the iron in the supplements, possibly mediated by increased duration of gestation and lower rates of preterm delivery. We cannot recommend that multiple micronutrients at the currently proposed dose^12^ should replace standard iron-folic acid supplements in pregnancy because they seem to provide less protection against preterm delivery and neonatal deaths in populations where iron deficiency in pregnancy is common. An adequate dose of iron is required in micronutrient supplements for pregnant women in developing countries to maximise reductions in neonatal mortality.

**What is already known on this topic**

Neonatal deaths remain a major cause of child mortality in those aged under 5 in developing countries and are associated with low birth weight
One of the major causes of low birth weight in developing countries is the poor nutritional status of the mother before and during pregnancy

**What this study adds**

Antenatal iron-folic acid increases the duration of gestation, reduces early preterm delivery, and is associated with a significant reduction in early neonatal mortality compared with folic acid alone
Antenatal multiple micronutrients modestly increase birth weight compared with folic acid but, despite this weight gain, are not associated with reduced early neonatal mortality
An adequate dose of iron is required in micronutrient supplements for pregnant women to maximise reductions in neonatal mortality in developing countries

## Notes

We thank all the women and their families who participated in the trial. We also thank the following members of the Shaanxi study team: Kang Yijun, Fang Bo, and Liang Weifeng (field supervisors); and the area coordinators of the Maternal and Child Healthcare Stations and Health Bureaus in the study counties. Ray Yip, Mary Cogswell, and Zuoguo Mei provided useful comments about the study design and field procedures. Li Qiang helped with data management. Wang Quanli, Xiao Shengbin, Xing Yuan, Zhou Xiaoyan, Wang Bei, and Xie Hong helped with data management. We also thank David Hipgrave, Sandy Huffman, Rachel Huxley, and Robyn Norton for valuable comments about the draft manuscript.

Contributors: All the authors contributed to the design and implementation of the study and reviewed drafts of the manuscript. LZ (coinvestigator in China) developed the study protocol, questionnaires, and field procedures, directed the field operations, held routine meetings with community leaders and health staff, contributed to the quality control, data cleaning, data analysis, data interpretation, and wrote the first draft of the paper. MJD (coinvestigator in Australia) developed the protocol, helped in the development of study questionnaires and field procedures, generated the cluster randomisation list, contributed to the data analysis and data interpretation, edited the paper, and is guarantor. YC contributed to the data management and quality control procedures and helped in training field staff. SD (coinvestigator in China) contributed to the preparation of the manual of operations and data interpretation. SC monitored the field operations, arranged the manufacture of supplements, kept the key for the treatment schedule, and contributed to data interpretation and preparation of the manuscript. LK monitored the field operations and contributed to data interpretation and preparation of the manuscript. HY was the principal investigator, developed the protocol, contributed to the questionnaires and field procedures, supervised the field operations, data analysis, and data interpretation, edited the paper, and is guarantor.

Ethical approval: This study was approved by the Human Research Ethics Committee of the College of Medicine, Xi’an Jiaotong University (No 2002001) and the Ministry of Health, China. All women gave informed verbal consent.

Provenance and peer review: Not commissioned; externally peer reviewed.

## Notes

**Cite this as: ***BMJ* 2008;337:a2001
